# Association Between Tea Drinking and Anemia in Women of Reproductive Age: A Cross-Sectional Study From the Mekran Division, Balochistan, Pakistan

**DOI:** 10.7759/cureus.64801

**Published:** 2024-07-18

**Authors:** Noman Sadiq, Yasmeen Gul, Muhammad M Bilal, Muhammad Afzal, Nasrin Mumtaz, Abdul Wahid

**Affiliations:** 1 Physiology, Mekran Medical College, Turbat, PAK; 2 Obstetrics and Gynecology, Mekran Medical College, Turbat, PAK; 3 Otolaryngology - Head and Neck Surgery, Mekran Medical College, Turbat, PAK; 4 Obstetrics and Gynecology, Jhalawan Medical College Khuzdar, Khuzdar, PAK; 5 Internal Medicine, Jhalawan Medical College Khuzdar, Khuzdar, PAK

**Keywords:** iron deficiency anemia, dietary habits, women of reproductive age, tea, anemia

## Abstract

Background: Anemia in females of the reproductive age group is an area of concern globally, but its prevalence is high in developing countries. Dietary habits and lifestyle impact the hematological parameters. It is important to evaluate the impact of tea drinking on hematological parameters in females of the reproductive age group.

Objective: The study aims to determine the association of tea drinking with anemia among women of reproductive age (WRA) in the Mekran division of Balochistan.

Methods: A cross-sectional observational study was conducted at Mekran Medical College (MMC), a teaching hospital in Turbat, Balochistan, by the Department of Obstetrics and Gynecology from December 2023 to May 2024. Using a non-probability convenience sampling technique, a total of 356 females, 16-35 years of age, were included in the study after getting informed consent. Pregnant females and those who were using any medication for anemia were excluded from the study. Blood samples were analyzed using a CBC hematology analyzer. Data were analyzed using SPSS 26 by applying an independent sample t-test and chi-square test.

Results: Among all 356 included participants, 193 females were anemic. Among the tea drinkers (n = 266), 159 participants were mild to severely anemic. While among non-tea-drinking women (n = 90), only 34 participants were mild or moderately anemic with no severe anemia. A significant association was found between tea drinking and anemia among WRA (p < 0.05). A significant mean difference was found in the hemoglobin (Hb), mean corpuscular volume (MCV), and mean corpuscular hemoglobin (MCH) levels among the tea-drinker and non-tea-drinker participants (p < 0.05).

Conclusion: The WRA group from the Mekran region is suffering from anemia. Women who drink tea suffer more from anemia. Effective healthcare strategies should be implemented to address the issue of anemia among WRA.

## Introduction

Anemia is defined as a reduced hemoglobin (Hb) concentration in blood, decreasing its overall oxygen-carrying capacity regardless of the underlying cause [1-2]. Non-pregnant women of reproductive age (WRA) with Hb concentrations <12 g/dL are classified as anemic. According to the WHO guidelines, anemia severity is categorized as mild (11-11.9 g/dL or 110-119 g/L), moderate (8-10.9 g/dL or 80-109 g/L), and severe (<8 g/dL or <80 g/L) [3].

Globally, there have been 1.8 billion cases of anemia, with a prevalence of 23,176.2 per 100,000 population, notably higher in South Asian, African, and Middle Eastern countries [2]. Among the estimated 378.3 million WRA residing in low- and middle-income countries (LMICs), 23.8 million cases occur in Pakistan [1]. The WHO identifies anemia prevalence ≥5% as a public health problem, categorized as mild (5-19.9%), moderate (20-39.9%), and severe (≥40%) [4].

Anemia can stem from micronutrient deficiencies, hemoglobinopathies, poor diet, parasitic infections, worm infestations, and chronic diseases, such as tuberculosis and HIV/AIDS [5]. Sociodemographic factors like age, financial status, education, BMI, health care utilization, marital status, birth spacing, parity, residence (urban/rural), hygiene, and infections contribute significantly to anemia prevalence among WRA in LMICs [6].

Iron deficiency anemia (IDA) is the most prevalent and preventable form worldwide among WRA. Reduced intake of iron-rich diets and increased physiological iron requirements during reproductive years heighten the risk of anemia [7]. Qadir et al. reported a high prevalence of iron-deficiency anemia among women under 30 in Quetta, Balochistan [8]. Chronic IDA in women is a leading cause of maternal and perinatal mortality, contributing to complications such as post-partum hemorrhage, congenital birth defects, small for gestational age, and low birth weight [9]. Mild preexisting anemia can worsen during pregnancy due to increased physiological demands, further elevating risks for maternal and neonatal complications [10].

Tea is widely consumed globally, especially in developing countries, but cultivation in contaminated soil can lead to heavy metal contamination. The literature suggests that tea consumption may contribute to IDA [11]. Anemia remains a persistent global health challenge, particularly in LMICs, prompting ongoing debates regarding its causes and associations, alongside established guidelines for prevention and treatment. Despite numerous studies on anemia prevalence and associated factors across Pakistan, limited literature exists from the Mekran region. This study aims to determine the prevalence of anemia among the WRA in the Mekran division and to explore the impact of tea drinking on anemia.

## Materials and methods

Study design and ethical approval

A cross-sectional observational study was conducted at Mekran Medical College (MMC), a teaching hospital in Turbat, Balochistan, by the Department of Obstetrics and Gynecology from December 2023 to May 2024. Ethical approval was obtained from the Ethical Review Committee of MMC, Turbat, via letter MMC/ERC/12/2023. Considering the prevalence of anemia as 30% in females of the reproductive age group, the sample size was calculated to be 323 with a 95% confidence interval (CI) using the OpenEpi software (OpenEpi: Open Source Epidemiologic Statistics for Public Health, www.OpenEpi.com) [12].

Inclusion and exclusion criteria

A non-probability convenient sampling technique was used to collect data. Females visiting the Department of Obstetrics and Gynecology were included in the study. The non-pregnant WRA (16-35 years) and not on the treatment for anemia were included in the study. Females with diagnosed hematological disorders, those who were on medication for anemia for any reason, and those who did not give consent for blood samples were excluded from the study.

Assessment of hematological parameters

Blood samples of 3 ml were collected by trained laboratory assistants following the standard protocol. A CBC hematology analyzer (Celltac Alpha MEK-6500, Nihon Kohden, Germany) was used for blood analysis. The anemia among participants was categorized according to the WHO guidelines into mild, moderate, and severe (WHO); based on the mean corpuscular volume (MCV), the results were classified as normocytic anemia (80-100 fL), microcytic anemia (<80 fL), and macrocytic anemia (>100 fL). Females who were consuming three or more cups of tea in a day were considered tea drinkers [11].

Data analysis

Data were entered and analyzed using IBM SPSS Statistics for Windows, version 26.0 (released 2019, IBM Corp., Armonk, NY). Qualitative variables were calculated in terms of frequencies. The mean difference of parameters among the tea drinkers and non-tea drinkers was calculated by applying an independent sample t-test. The association of tea drinking with anemia was calculated by applying the chi-square test. A p-value < 0.05 was considered significant.

## Results

The data from the 356 participants were included in the study. The mean age of the participants was 27.39 ± 5.30. The sociodemographic data of the study participants including their residence, family type, education, and tea-drinking status are shown in Table 1.

**Table 1:** Sociodemographic data of the study participants. The data are presented as %.

Age in years (mean + SD)	27.39 + 5.30 (N = 356)
Residence	
Rural	145 (40.7%)
Urban	211 (59.3%)
Family type	
Nuclear	41 (11.5%)
Combined	315 (88.5%)
Educational status	
Illiterate	245 (68.8)
literate	111 (31.2%)
Tea	
Drinkers	266 (74.72%)
Non-drinkers	90 (25.28%)

Among all the study participants, 54.21% (193) were anemic. Among the tea drinkers, more than half were anemic. Overall, a significant association was found between anemia and tea drinking (p < 0.001). Among all the study participants, 44.38% (158) were suffering from IDA. However, 82.91% (131) of the participants suffering from iron deficiency anemia were consuming tea. A strong significance was found between tea drinking and microcytic anemia (p = 0.002) (Table 2).

**Table 2:** Significant association of tea drinking with anemia among women of reproductive age. Chi-square test was applied. *p < 0.05.

Variable	Tea drinkers (n = 266)	Non-tea drinker (n = 90)	Chi-square X^2^	p-value
Non-anemic	107 (30.1%)	56 (15.7%)	19.69	<0.001*
Mild anemia	105 (29.5%)	31 (8.7%)		
Moderate anemia	51(14.3%)	3 (0.8%)		
Severe anemia	3 (0.8%)	0 (0%)		
Non-anemic	107 (30.1%)	56 (15.7%)	19.14	0.002*
Mild microcytic anemia	81 (22.8%)	24 (6.7%)		
Mild normocytic anemia	24 (6.7%)	7 (2%)		
Moderate microcytic anemia	47 (13.2%)	3 (0.8%)		
Moderate normocytic anemia	3 (0.8%)	0 (0%)		
Severe microcytic anemia	3 (0.8%)	0 (0%)		

The mean values of the hemoglobin level, MCV, and mean hemoglobin concentration (MHC) were significantly reduced in participants drinking tea as compared to participants who were not consuming tea (p < 0.001). The detailed hematological parameters among the tea drinkers and non-tea drinkers are given in Table 3.

**Table 3:** Significant reduction of hemoglobin, MCV, and MCH levels in women drinking tea. An Independent sample t-test was applied. *p < 0.05. WBCs: white blood cells,  RBCs: red blood cells, Hb: hemoglobin, Hct: hematocrit, MCV: mean corpuscular volume, MCH: mean corpuscular hemoglobin, MCHC: mean corpuscular hemoglobin concentration, PLT: platelets, LYM: lymphocytes, NEUT: neutrophils

Parameter	Tea drinkers Mean + S.D (n = 266)	Non-tea drinkers Mean + S.D (n = 90)	p-value
WBCs (per mm^3^)	8.14 + 3.49	9.45 + 9.92	0.066
RBCs (million/mm^3^)	4.81 + 1.63	5.06 + 3.73	0.385
Hb (g/dl)	11.41 + 1.55	12.16 + 1.27	<0.001*
Hct (%)	37.47 + 7.11	37.19 + 6.85	0.908
MCV (μm^3^)	74.95 + 10.21	78.05 + 10.09	0.013*
MCH (pg)	25.41 + 6.45	27.66 + 8.37	0.016*
MCHC (%)	32.65 + 17.58	35.90 + 13.05	0.237
PLT (10^9^/L)	290.58 + 76.8	299.54 + 92.27	0.365
LYM (%)	34.72 + 20.56	32.20 + 12.26	0.301
NEUT (%)	51.59 + 14.16	54.43 + 14.62	0.129

## Discussion

Anemia has become a global health challenge, particularly in women in LMICs. Among the 10 countries of South and Southeast Asia (SSEA), including Pakistan, anemia prevalence among WRA is 50.17%, ranging from the lowest in the Philippines to the highest in Nepal. Factors contributing to this include younger age, poor financial status, lower educational levels, and inadequate dietary nutrition [13].

In Pakistan, an average of 46.5% of WRA are anemic [14]. Microcytic anemia is the most prevalent type among them. Factors contributing to anemia include women's age, irregular meal times, multiple pregnancies, and daily tea consumption [15]. In addition, the smokeless tobacco "Gutka" is significantly associated with anemia among women in Karachi, Pakistan [16]. In rural Pakistan, low meat intake and breastfeeding are significant factors associated with lower iron and ferritin levels among anemic women [17].

This study found a significant association between tea drinking and anemia among WRA. It also showed significant mean differences in hemoglobin levels, MCV, and MCH between tea drinkers and non-tea drinkers. Tea drinking is linked to the occurrence of microcytic anemia in women, consistent with prior research in Pakistan indicating a higher prevalence of anemia, lower hemoglobin levels, and reduced red cell count among tea drinkers compared to non-tea drinkers [11]. Similar findings of anemia were observed among female university students in Pakistan who consume tea [18]. A systematic review and meta-analysis further revealed that habitual daily tea drinkers are at a 1.94 times higher risk of developing anemia [19].

Iron is mainly absorbed in the non-heme form in the human gut. Polyphenol compounds found in black tea significantly inhibit the absorption of free iron in the duodenum, potentially increasing the susceptibility of tea drinkers to developing iron deficiency and anemia [20]. Controlled trials have shown that tea's polyphenols notably decrease fractional iron absorption in women, thereby reducing iron bioavailability [21]. The common preparation of black tea with milk, known as "chai," further reduces the absorption of free iron from the gut due to the complex formation between milk proteins and polyphenols, which decreases iron solubility and may contribute to iron deficiency [22]. By contrast, a study in Korea found no significant correlation between green tea consumption and ferritin levels among women, nor was there an association between green tea consumption and participants' iron status [23].

Iron supplementation has proven effective in enhancing the iron profile and alleviating anemia among WRA. However, it's crucial to consider dietary factors that can reduce iron absorption, such as tannins, calcium, and phytates. Strategies to counteract reduced iron absorption include consuming enhancers like ascorbic acid, meat, and acidic foods alongside iron supplements [16,21]. Fortified foods containing iron salts have demonstrated improvements in iron profiles by enhancing iron absorption in anemic WRA [24]. Nevertheless, black tea has been shown to decrease iron absorption from fortified food items, which could potentially undermine the effectiveness of food fortification programs aimed at combating anemia [21].

Healthcare providers can implement an effective anemia prevention plan through education and supplementation to safeguard WRA from anemia [25]. Given that anemia is a multifactorial issue, a comprehensive approach is essential. While our study sheds light on the potential association of tea consumption with anemia among WRA in the resource-constrained underdeveloped region of Mekran, Pakistan, it is important to acknowledge its limitations. We relied solely on CBC parameters to assess anemia, which may not capture the exact causal relationships and other influencing factors. Future research should consider evaluating additional social, environmental, hormonal, and medical factors to provide a more comprehensive understanding of anemia in this population. Despite these limitations, our findings contribute valuable insights into a largely understudied area, informing potential avenues for targeted interventions and healthcare strategies.

## Conclusions

WRA from the Mekran region are suffering from anemia. Tea drinking enhances anemia among WRA. To mitigate this health issue, it is crucial to implement effective healthcare strategies tailored to the needs of this population. These strategies should encompass comprehensive screening programs, nutritional interventions, and community education initiatives. By addressing the factors contributing to anemia, such as dietary habits and access to healthcare, we can improve the overall health and well-being of women in the Mekran region.
